# EFFICACY AND SAFETY OF KNOTLESS BARBED SUTURES IN CAPSULAR CLOSURE FOLLOWING DISTAL FEMUR FRACTURE FIXATION

**DOI:** 10.1590/1413-785220233101e250368

**Published:** 2023-04-17

**Authors:** Amit Lakhani, Kavin Khatri, Neeraj Malhotra, R.K Banga, Deepak Bansal

**Affiliations:** 1 Dr B R Ambedkar State Institute of Medical Sciences, Mohali, Punjab, India.; 2 All India Institute of Medical Sciences, Department of Orthopaedics, Bathinda, Punjab, India.; 3 Government Medical College, Department of Orthopaedics, Amritsar, Punjab, India.; 4 Government Medical College, Department of Orthopaedics, Patiala, Punjab, India.; 5 AIMC Bassi Hospital, Department of Orthopaedics, Ludhiana, Punjab, India.

**Keywords:** Surgical Procedure, Suture Techniques, Femur, Femoral Fractures, Procedimento Cirúrgico, Técnicas de Sutura, Fêmur, Fraturas Femorais

## Abstract

**Introduction:**

Good wound closure is an important step in management of distal femur fracture to prevent infection and faster rehabilitation. Knotless barbed sutures can save time and distribute wound tension evenly. However, its role in terms of functional outcome, closure time, and postoperative complications has not been studied in a distal femur fracture.

**Material and methods:**

A total of 47 patients aged more than 18 years of distal femur fracture treated with distal femur locking plate were randomized either into either barbed or traditional suture groups. in the barbed group, capsular wound closure was carried out with 2-0 bidirectional barbed knotless sutures (Quill SRS^®^ PDO, Angiotech, Vancouver, BC, Canada). In patients assigned to group B, capsular closure was done with 1-0 Vicryl^®^ (Ethicon inc. Somerville, NJ) and 5-0 Ethibond^®^ alternatively.

**Results:**

The mean flexion at the knee joint was 105.7±15.6 degrees in the study group while it was 110.4±13.7 in the control group (p= 0.2133). Mean estimated closure time was significantly shorter in the study group as compared to the control group (p<0.05). Cases of needle prick injury were higher in traditional suture group. Patients developed stitch abscess and superficial infection in both groups. However, the difference in incidence between the two was not statistically significant

**Conclusion:**

Barbed suture is an efficient method of wound closure. It reduces wound closure time with similar complication rate as with use of conventional sutures. ***Evidence Level II; Randomized Clinical Trial.***

## RESUMO

**Introdução:**

O fechamento adequado da ferida é um passo importante no manejo da fratura distal do fêmur a fim de evitar infecção e permitir uma rápida reabilitação. Suturas farpadas sem nós podem poupar tempo e distribuir uniformemente a tensão da ferida. Entretanto, seu papel em termos de resultado funcional, tempo de fechamento e complicações pós-operatórias não tem sido analisado em casos de fratura distal do fêmur.

**Material e métodos:**

Um total de 47 pacientes com mais de 18 anos de idade com fratura distal do fêmur tratados com placa de fixação distal do fêmur foram aleatorizados em grupos de sutura farpada ou tradicional. No grupo de farpados, o fechamento da ferida capsular foi feito com suturas sem nós farpados bidirecionais 2-0 (Quill SRS^®^ PDO, Angiotech, Vancouver, BC, Canadá). Em pacientes designados para o grupo B, o fechamento capsular foi feito com Vicryl^®^1-0 (Ethicon inc. Somerville, NJ) e Ethibond^®^ 5-0 respectivamente.

**Resultados:**

A flexão média na articulação do joelho foi de 105,7±15,6 graus no grupo de estudo e 110,4±13,7 no grupo controle (p= 0,2133). O tempo médio estimado de fechamento foi significativamente menor no grupo de estudo em comparação com o grupo controle (p<0,05). Os casos de ferimento por perfuração da agulha foram maiores no grupo de sutura tradicional. Os pacientes desenvolveram abscesso de pontos e infecção superficial em ambos os grupos. Entretanto, a diferença na incidência entre os dois não foi estatisticamente significative

**Conclusão:**

A sutura farpada é um método eficiente para o fechamento de feridas. Ele reduz o tempo de fechamento das feridas com uma taxa de complicação semelhante à utilização de suturas convencionais. ***Evidência Nível II; Ensaio Clínico Randomizado.***

## INTRODUCTION

The incidence of distal femoral fracture is approximately 10 per 100,000 with bimodal age distribution.^1^ Despite continued improvements in the implants used for fixation of distal femur fractures, stiffness is one the most commonly encountered problem. Stiffness results from delayed mobilization at the knee joint following fracture fixation in the majority of cases.^2,3^ With the invent of newer distal femur locking plates active mobilization at knee joint is possible in the immediate postoperative period. However, there are other factors like closure of arthrotomy wound and suture material used which could determine the end results. Traditionally, arthrotomy closure with simple interrupted sutures and multiple knots results in uneven tension and is also time consuming. From this perspective, knotless sutures allow multiple simultaneous bidirectional knots distributing tension evenly across the entire length of arthrotomy and also saves valuable time.

Barbed sutures are being used in urogynaecological procedures, general surgery and plastic surgery.^4-6^ However, there is debate over the safety profile of knotless barbed sutures across the spectrum. Majority of the studies carried out till date evaluated for closure time, functional outcome and complications in arthroplasty procedures.^7-9^ To the best of our knowledge, no study to date had evaluated complication rate and functional outcome of capsular closure carried out with barbed knotless sutures in distal femur fracture fixation. The aim of study was to determine if barbed sutures offer any advantage over conventional sutures in case of arthrotomy closure following distal femur fracture fixation. We hypothesized that use of barbed knotless sutures would result in 1) shorter wound closure time 2) similar complication rates 3) better clinical outcomes in comparison to traditional knotted sutures as early mobilization could be initiated at the knee joint.

## MATERIAL AND METHODS

The current study is a randomised controlled trial comparing two different techniques of wound closure (barbed knotless versus standard knotted sutures) in cases of distal femur fracture treated with locking compression plate. The approval for this study was obtained from the institutional review board. The consent was taken from the patients prior to surgery.

Patients included in the study were cases of distal femur fracture (AO type 33 A, B, C1 and C2) treated with single distal femur locking plate operated through lateral parapatellar approach. Complex distal femur fracture AO type 33C3 were not included in the study as they would require sometimes dual plating or medial parapatellar approach for management of fracture. Patients with Gustilo Anderson type 2 and 3 compound injuries, multiple injuries, head injury, subjects below the age of 18 years and patients with uncontrolled diabetes mellitus were also excluded from study.

The study was powered to calculate the number of participants necessary to detect a difference of five minutes between two suture groups from previous studies conducted in use of barbed sutures.^8^ With an alpha of 0.05 and a power of 80%, we expected the findings to be significant if the number of subjects was 47.

Patients were randomly assigned to receive closure using barbed knotless sutures (Group A) or standard conventional sutures (Group B). Randomisation process was performed with the closed envelope system just before starting the closure process. Previous to the study, all the participants went through training with application of barbed knotless sutures in not less than 10 cases. Participants were not informed of their allocation during the trail but they could request for the information at the end of study. Surgeons and supporting staff were not blinded. Though radiographs were reviewed by independent assessors still due to presence of implants they were not blinded.

Subjects were reviewed in daily trauma meet and operated on next available theatre. Each patient received a single intravenous dose of cefuroxime preoperatively one hour prior to surgery and two intravenous doses postoperatively. Preparation, fracture reduction and other intraoperative decisions were left to the discretion of the operating surgeon. The distal femur fractures were approached through lateral parapatellar knee arthrotomy. All fractures were fixed with the help of distal femur locking compression plate (Nebula Surgical, Rajkot, India) plate. All surgeries were performed in an inpatient setting with variable length of stay depending upon pain and functional ambulatory status of the patient.

In patients assigned to group A, capsular wound closure was carried out with 2-0 bidirectional barbed knotless sutures (Quill SRS^®^ PDO, Angiotech, Vancouver, BC, Canada). Barbs are arranged in a helical fashion around the suture radiating in both directions.^10^ They created a tension in the opposing direction when passed through tissue working towards ends starting at the midpoint of wound. The operating surgeon worked towards one end while the assistant proceeded towards the other end. At the ends, the suture direction was reversed and again approached towards the midpoint. After engaging a few throws, the suture ends were cut without knot tying. In patients assigned to group B, capsular closure was done with 1-0 Vicryl^®^ (Ethicon inc. Somerville, NJ) and 5-0 Ethibond^®^ alternatively. The skin and subcutaneous tissue were closed with the help of skin staplers and Vicryl 2-0 respectively in both groups. The capsular closure was carried out by the operating surgeon while skin and subcutaneous closure was completed by the assistant. The total closure time i.e. period of commencement of first stitch to skin closure was registered in both groups with the help of a stopwatch. The stopwatch was stopped in case of suture breakage and resumed once the new suture pack was opened again.

Non-adherent primapore dressing was applied over the wound. Dressing was changed on the second postoperative day and subsequently after every three days till suture removal at 14th postoperative day.

The in-bed mobilisation of the patients was started on the first postoperative day. They were advised to bend the knee as per pain tolerance. Toe touch and partial weight bearing was advised as per progress of fracture healing and stability. Full weight bearing was allowed between 9 to 12 weeks upon fracture union.

Preoperative data was collected on a standard form at the time of admission. Operative parameters included were start time of wound closure, completion of wound closure, total number of sutures used and any other intraoperative complication. Wound infection was graded based on the scale described by Hollander et al^11^ It was graded as no infection, simple stitch abscess, cellulitis, accompanying lymphangitis and systemic symptoms.

The functional outcome was evaluated using EQ-5D-5L^12^. EQ-5D- 5L is a health utility instrument to measure quality of life across five domains namely mobility, self-care, usual activities, pain and depression. It was assessed after operative procedure, at the end of 6 weeks and 12 weeks. Radiographs were evaluated on the second postoperative day, at six weeks and at 12 weeks. Loss of fixation, varus or valgus deformity of greater than 5 degrees, shortening of more than one centimetre, recurvatum/ procurvatum >10 degree were recorded.

### Statistical analysis

Demographic technical and risk factors were compared between both the groups to detect the confounding effects. Means with standard deviation were reported for all continuous variables and compared using independent 2-tailed t tests. Categorical variables were reported as frequencies per population and compared by chi-square analysis. Fisher›s exact test was used when in groups with five or fewer subjects. Spearman's correlation coefficient was used to determine if there was association between different variables in the study group. The p-value for describing statistical significance was set at <0.05.

## RESULTS

**Figure 1:**
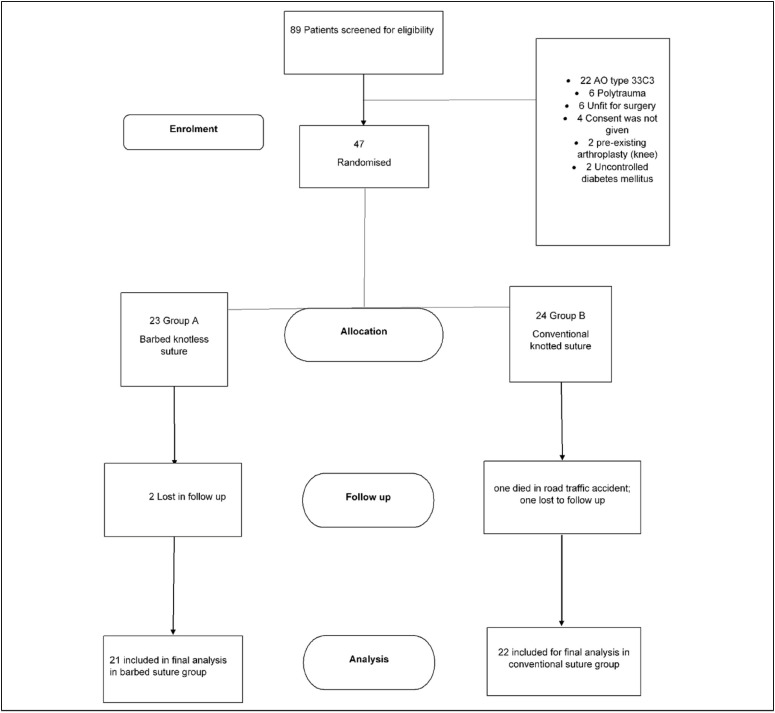
Consolidated standards of reporting trails (consort) flow diagram.

Patients were recruited between September 2014 and November 2020. Of the 89 patients screened, the most common reason for not inclusion of subjects in study was AO type C3 of distal femur fracture. (Figure 1) 47 participants were randomised into the trail. The study group consisted of 23 cases while the knotted/control group comprised 24 cases. There were 14 females (29.78%) and 33 males (70.21%). Patient characteristics including age, gender, body mass index and smoking status were recorded (Table 1).

**Table 1:** Patient Demographics.

Variable	Barbed group (n=23)	Knotted suture group (n=24)	P value
Age (years)	43.2 8.7	41.2 6.7	0.3807
**Gender**			
Male	17 (74%)	16 (67%)	
Female	6 (26%)	8 (33%)	
Body Mass Index (kg/m^2^)	20.4 7.3	22.3 5.6	0.3209
Smoker	3 (13%)	2 (8%)	0.5794
Controlled Diabetes	4 (17%)	2 (8%)	0.3546
**Mechanism of injury**			
Road Traffic Accident	18 (78%)	16 (67%)	
Fall while walking	5 (22%)	8 (33%)	
Time interval from admission to surgery (days)	5 2.4	6 2.7	0.1870
**Fracture Classification (AO/OTA)**			
A1	7 (30.4%)	6 (25%)	
A2	2 (8.7)	2 (8.3%)	
B1	1 (4.3%)	2 (8.3%)	
B3	0 (0%)	1 (4.1%)	
C1	8 (34.7%)	7 (29.1%)	
C2	5 (21.7%)	4 (16.7%)	

Mean estimated closure time was significantly shorter in the study group as compared to the control group (p<0.05). The incidence of suture breakage was significantly higher in the group using barbed knotless sutures for wound closure (p=0.02). The surgical staff had reported no case of needle stick injury in the barbed suture group while three cases were reported in the conventional suture group. The difference was not statistically significant (p=0.08). (Table 2) The mean flexion at the knee joint was 105.7±15.6 degrees in the study group while it was 110.4±13.7 in the control group (p= 0.2133). The difference was not clinically significant.

**Table 2:** Outcome by type of suture.

Variable	Barbed suture group (n=23)	Conventional suture group (n=24)	P value
Wound closure time (in minutes)	11.5 3.4	17.3 4.5	< 0.0001
Suture breakage	5 (20.8%)	0	0.02
Needle prick	0	3 (12.5%)	0.083

Among the 47 subjects, EQ-5D-5L questionnaire was completed by 41 (87%, 95%CI 65% to 97%), 30 (65%, 95%CI 43% to 83%) and 29 (61%, 95%CI 39% to 80%) patients at the baseline, 6 weeks and 12 weeks. The data collected was insufficient to achieve any meaningful conclusion between the groups. (Table 3)

**Table 3:** Patient reported outcome measured through EQ-5D-5L heath questionnaire.

Time frame	Barbed suture group (n=23)	Conventional suture group (n=24)
Post injury	-0.04 (0.26;11)*	-0.05 (0.25;10)
6 weeks	0.35 (0.32; 9)	0.19 (0.17; 7)
12 weeks	0.40 (0.37; 8)	0.36 (0.31; 9)

* Indicates mean; standard deviation.

### Wound complications

Patients developed stitch abscess, superficial infection and lymphangitis in both groups. However, the difference in incidence between the two was not statistically significant. (Table 4) One patient in the barbed suture group had developed deep seated infection and was subjected to irrigation and debridement. Intravenous antibiotics (injection piperacillin with tazobactam) for six weeks followed by oral antibiotic therapy (tablet cefuroxime) were administered. There was no recurrence at the time of last follow up (one and half years).

**Table 4:** Wound complications.

Variable	Barbed suture Group (n=23)	Conventional suture group (n=24)	P value
Stitch abscess	4 1.8	3 1.9	0.0708
Cellulitis	3 1.1	3 1.3	1
Sepsis with systemic symptoms	1 0.5	0	>0.99
Lymphangitis	1 0.5	1 0.5	1

Cellulitis was analysed to assess for association between dermal closure (r = -0.03, 95% CI -0.14 to 0.09), smoking (r = 0.01, 95% CI -0.08 to 0.11), age (r = 0.02, 95% CI -0.09 to 0.12) and BMI (r = -0.06, 95% CI -0.05 to 0.17), none of were found to be correlate at six weeks.

Subsequently, two tailed post hoc analysis was carried out to determine the number of distal femur fractures required to get a statistically significant difference in cellulitis and incidence of needle stick injury (power at 0.8 and ∝ = 0.05). It was calculated that the number of subjects in each group required to detect any statistically significant result would be 418,321 for cellulitis and 819 for needle stick injury. So, it is likely that no clinically significant between the two group exists.

## DISCUSSION

Good wound closure is critical to minimize wound related complications.^13^ The principle finding of the current study is that barbed knotless sutures significantly reduce the closure time following fixation of distal femur fracture when compared with conventional sutures. Another finding of the study was that the complication rates were similar in both groups and do not depend upon the type of suture material used.

Wound closure was faster in a barbed knotless suture group and similar findings were noted in other studies though conducted in arthroplasty patients. Chan et al^10^ reported an average reduction of 4 minutes in closure time whereas Gilliland et al^14^ noted a reduction of 4.6 minutes in overall closure time. Such minor reduction in surgical time needed for closure of an arthrotomy wound does not have any repercussions on the long-term results.

Interrupted knotted sutures have traditionally been used in closure of arthrotomy wounds following fixation of distal femur fractures The conventional interrupted sutures have few disadvantages. Handling of needle during knot tying puts surgeons at an additional risk of injury. Interrupted sutures put uneven pressure along the length of wound which might lead to tissue ischemia resulting in necrosis in some cases and resultant tissue could be a source of infection. Knotless barbed sutures however had several advantages like equal distribution of tension across the length of wound and minimal risk to surgeons due to lesser knot tying. The wound healing related complications were not significantly different in two groups. There was also no major difference in satisfaction scores. Range of motion at the knee joint, especially flexion showed no difference between barbed knotless sutures and traditional sutures. The similar finding was noted by Chan et al.^10^ We had expected better results with barbed knotless suture because running suture share out mechanical forces in a better way. However, there are many other factors which affect the range of movement at the knee joint.

It is also worthwhile to note that the barbed sutures resist failure to a greater extent as compared to conventional sutures. In case of suture rupture, the anchoring barbs hold the suture in a place. Vakil et al^15^ in their study on cadaveric knees used barbed sutures for closure of arthrotomy wounds and subjected to repeated cycling. It was concluded that the arthrotomies closed with barbed sutures resisted failure in comparison to conventional sutures. However, there are higher chances of suture breakage as encountered in our study. There were five cases of suture breakage in the barbed group in comparison to the control group and the difference was statistically significant (p=0.02).

Morris et al^16^ suggested that barbed monofilament sutures were associated with decreased bacterial adhesion in comparison to conventional suture. This should hence protect against infection. However, Campbell et al^17^ and Chawla et al^18^ reported higher incidence of infection with use of barbed sutures. In our study there was no difference between the barbed suture and conventional suture application. There were limitations to our study. First, with a limited number of subjects we could not ascertain if there is statistically significant difference between groups in rates of wound related complications and needle stick injuries. However, with the large group and post hoc analysis, clinically significant difference in wound related complications could not be ascertained. Second, many confounding variables like obesity and comorbid conditions could be excluded before randomization which may have an effect on final outcome. Third, the study involved cases of distal femur fracture and hence the findings of the same cannot be extrapolated for its use in other orthopaedic procedures.

In conclusion, use of barbed sutures is associated with shorter closure time, a higher chance of suture breakage and similar functional outcome. There were similar wound-related closure complications in comparison to conventional closure of arthrotomy wounds after distal femur plating.
